# An algae-based polymer material as a pesticide adjuvant for mitigating off-target drift

**DOI:** 10.1016/j.heliyon.2024.e35510

**Published:** 2024-08-02

**Authors:** Narayanan Kannan, Quentin Read, Weiqiang Zhang

**Affiliations:** a Pollinator Health in Southern Crop Ecosystem Research Unit, United States Department of Agriculture-Agricultural Research Service, Stoneville, MS, 38776, USA; b Southeast Area, United States Department of Agriculture-Agricultural Research Service, Raleigh, NC, 27695, USA

**Keywords:** Sodium alginate, Pesticide adjuvant, Polyacrylamide, Pollinators, Honeybee, Cotton

## Abstract

Off-target pesticide drift from cropland is a major source of pesticide exposure to pollinating insects inhabiting crop and wildlands in the lower Mississippi Delta (LMD) in the USA. This study is aimed to develop a drift-reducing pesticide adjuvant that is less/nontoxic to honeybees. Ongoing toxicology experiments with two widely-used insecticides and sodium alginate (SA) pointed out reductions in honeybee mortality compared to an industry standard reference polyacrylamide (PAM). When used as an adjuvant to spray the same insecticides described above, SA did not interfere in killing the target pests. Therefore, SA has been tested as a drift-reducing pesticide adjuvant to protect honeybees. Spray experiments in the lab were carried out in four sets: (i) water only, (ii) water and adjuvant, (iii) water and pesticide, and (iv) water, pesticide and adjuvant. Each set contained 18 treatment combinations to cover the ranges in spray pressure (three), adjuvant dose (three), and spray nozzles (two). The droplet spectrum was analyzed using a P15 image analyzer. Diameters of 10 %, 50 % and 90 % volumes (DV_10_, DV_50_, and DV_90_), droplet velocity, standard deviation and relative span were measured. The drift reduction potential (DRP) of SA was analyzed by (i) dose, (ii) spray pressure, and (iii) nozzle type. The DRP of SA is compared to that of PAM. Additionally, three field experiments were carried out to analyze the efficiency of SA in reducing pesticide drift. The results from our experiments collectively indicate that SA has significant potential in mitigating drift as well as minimizing pesticide toxicity to honeybees.

## Highlights

• Pollinators are exposed to drift from pesticide products including adjuvants.
• We tested the drift reduction potential of an algae-based adjuvant.
• Our laboratory and field experiments suggested reductions in off-target drift.
• The new adjuvant is less toxic to honeybees and did not interfere in killing pests.

## 1 Introduction

The lower Mississippi Delta (LMD) is one of the most productive agricultural regions in the USA where intensive agricultural activities are the norm. Soybean, cotton and corn are the major crops cultivated in the region [1]. Profitable commercial-scale crop production in intensive agricultural systems such as those in LMD inevitably involves the use of pesticides. Diversity and abundance of native pollinators, managed honey bees and the bee keeping industry are simultaneously booming in the region [1], as the LMD offers a unique ecoregion for biodiversity. However, the health of pollinators in this region is likely to be compromised largely due to exposure to toxic chemicals resulting from pesticide drifts from spraying events across cropped areas [[2], [3], [4], [5]]. Apart from the active ingredient that targets the pest (insects/weeds/fungi), several other chemicals are added in the pesticide tank mix to make the application more efficient. The functionalities of the added chemicals include reduction of off-target pesticide drift, increased ability of pesticide to retain to the target surface, penetration into the leaf surface and incorporation of active ingredient into the plant.

Adjuvants known to improve pesticide application efficiency and minimize off-target drift are widely used in pesticide applications [[6], [7], [8], [9], [10], [11]]. The Council of Producers and Distributors of Agrotechnology (CPDA) certified 221 adjuvants as approved tank mix products (adjuvant products with CPDA certification, https://cpda.com/cpda-certified-product/) [12]. Many adjuvant products that are not certified by CPDAs are also commonly used by commercial-scale producers, although their effectiveness is not well known.

Studies on pollinator safety show that some adjuvants are equally or more toxic than the pesticide active ingredients [[13], [14], [15]]. Therefore, in this study we aimed to develop a pesticide additive less toxic or nontoxic to the pollinators that can act as an adjuvant to apply pesticide to the target surface and mitigate off-target drift. The proposed adjuvant material is based on widely available and inexpensive alginates, which are natural linear polysaccharides produced from brown algae (Phaeophyceae, one of the major seaweeds of temperate and polar regions) [16]. Alginates are anionic polymers; the most commercially available form is sodium alginate (SA) [17,18].

Off-target drift depends on factors such as weather, application equipment and type of chemical sprayed. High temperature and low humidity results in faster evaporation of the chemical during and after application and favors vapor drift that can be carried out to long distances [19]. Wind speed is also important. Putting together the factors on chemical and spraying equipment, the droplet size [20], spray pressure, velocity of spray particles [20], spray height [21] determines the application efficiency and off-target drift. Smaller droplets have better retention on target surface (such as leaf) but suffer more drift potential. Larger droplets on the other hand have poor retention but low drift potential [22]. Therefore, striking a balance is the key to improve efficient pesticide application and reduce off-target drift. When beekeeping is practiced in areas adjacent to cropped fields, we need to take that into consideration during chemical application. Pesticide adjuvants that can increase viscosity, make larger spray droplets, improve retention on target surface and help to reduce off-target drift. Polymers such as SA when added to pesticide tank mix have the potential to increase droplet size and reduce drift. Particles with more spray velocity reach the target sooner and reduce the opportunities to drift away. The pesticide adjuvants depending on their chemical characteristics may or may not help to improve spray velocity.

To protect the beekeeping industry and insect pollinators, there is an urgent need to develop and use nontoxic adjuvants. It can be envisioned that adjuvants having the following properties will have an enormous positive impact on the pesticide market and receive encouragement from redefined policies on pesticide application: (i) compatible with the commonly used herbicides, fungicides, and insecticides, (ii) not affecting the pesticide efficacy on the target crop pests, and (iii) not having adverse effects on beneficial insects such as bees and insect pollinators. Here, we report our results on the drift reduction ability of SA.

## 2 Materials and methods

### 2.1 Adjuvant materials

Sodium alginate (SA) (C_6_H_7_ O_6_Na)_n_ with a molar mass of 1.93 × 10^5^ g/mol is nontoxic, biocompatible, and biodegradable [17]. It is nearly odorless and tasteless [23], [16]. The solubility of SA in water is pH dependent. When pH is less than 3.4, SA becomes insoluble. After dissolving in water, it tends to form a thick colloidal solution with increasing concentrations. SA is insoluble in most organic solvents. Depending on the grade/resources, the viscosities vary from 20 to 400 cP for a 1 % solution at 20 °C. SA is listed by the United States Food and Drug Administration [24] as a “Generally Recognized as Safe” (GRAS) material. In the European Union (EU), it is registered as a food improvement agent [25]. The United States Environmental Protection Agency (USEPA) rated SA as a safer choice chemical [26]. SA is used in drug delivery systems and as a wound dressing material in pharmaceutical and medical fields [[27], [28], [29]].

In the food industry, SA is used as a texturizer, stabilizer, firming agent, and flavor adjuvant [30]. In water and wastewater treatment it is used as a flocculant. In other industries, it is used as surface active agent, processing aid, emulsifier, drilling mud and a formulation aid. To our knowledge, this is the first time it has been attempted as a drift-reducing agent for pesticide application to protect pollinators.

In current industry, one of the commonly used standard adjuvants is polyacrylamide (PAM). The drift-reducing capabilities of SA will be compared against PAM in our study. PAM [-CH_2_CH(CONH_2_)-]n (https://polymerdatabase.com/main.html) is a nonionic, water soluble, and biocompatible polymer. The largest use of PAM is in the water and wastewater treatment industry as a flocculant [31,32]. It is extensively used in soil conditioning and erosion control [[33], [34], [35], [36]] and in the oil and gas industry [37]. Some previous research has documented that high levels of acrylamide cause cancer in laboratory animals and is reasonably anticipated to be a human carcinogen [[38], [39]]. However, the risk posed by acrylamide compounds to humans is not fully understood. This reiterates the importance of the current research to minimize the drift of pesticide mixes to humans and other animals, including pollinators in the environment which are an important part of our food production system.

### 2.2 Physicochemical characteristics of the adjuvant materials

Millipore pure water was used as a reference to compare the physicochemical characteristics of the adjuvant solutions at different concentrations. A pH electrode and meter (Thermo Scientific, Chelmsford, MA, USA) were used to estimate the pH values of the solutions. Standard buffer solutions of pH 4.01, 7.01 were used to calibrate the electrode. Density was measured using a VWR specific gravity hydrometer (Radnor, PA, USA). The viscosity values were measured using an IKA Rotavisc lo-vis control viscometer (IKA-Werke GmbH & Co. KG, Staufen, Germany) with spindle SP1 at 22 °C. The viscometer was calibrated using the certified standard silicon oil. A KRÜSS bubble pressure tensiometer BPT100 (KRÜSS GmbH, Hamburg, Germany) was used to estimate the dynamic surface tension values of the adjuvant solutions and water. The measurements of pH, density, and dynamic surface tension measurements were carried out at 23 °C. All the estimated physicochemical properties of the adjuvant solutions are presented in Table 1.

**Table 1:** Physicochemical characteristics of water and different adjuvant solutions.

Item	Appearance	pH	Density (g/cm^3^)	Dynamic Viscosity (mPas)	Dynamic Surface tension (mN/m)^a^
Millipore pure water Sodium alginate 1.25 g/L Sodium alginate 2.5 g/L Sodium alginate 5.0 g/L Polyacrylamide 75 μg/L Polyacrylamide 187 μg/L Polyacrylamide 300 μg/L	Clear liquid Nearly a clear solution Nearly a clear solution Nearly a clear solution Homogeneous milky suspension Homogeneous milky suspension Heterogeneous milky suspension	7.00 6.86 6.92 6.99 5.83 5.99 6.02	0.9968 0.9968 0.9971 0.9973 0.9969 0.9970 0.9972	3.47 5.70 7.53 11.15 3.93 4.33 3.90	75.3–74.9 76.0–75.0 76.0–75.0 76.5–75.0 74.8–70.1 74.9–62.8 74.5–55.7

a Values represent surface ages of 10 ms–100,000 ms.

Freeze‒Thaw experiments were also performed to analyze the applicability of the adjuvant polymer in pesticide applications in colder climates and to analyze the ability to store them at low temperatures. Freeze‒thaw experiments were carried out three times with five trials in each experiment. In each experiment, the polymer solutions were subjected to five days of continuous freeze‒thaw cycles. On each day of the experiment, the solutions were allowed to freeze at −6.7 °C (20 °F) for 6 h and allowed to thaw overnight before the next freezing experiment. After each freeze/thaw experiment, the solutions were photographed to visually look how they appeared.

### 2.3 Experimental design

**Fig. 1:**
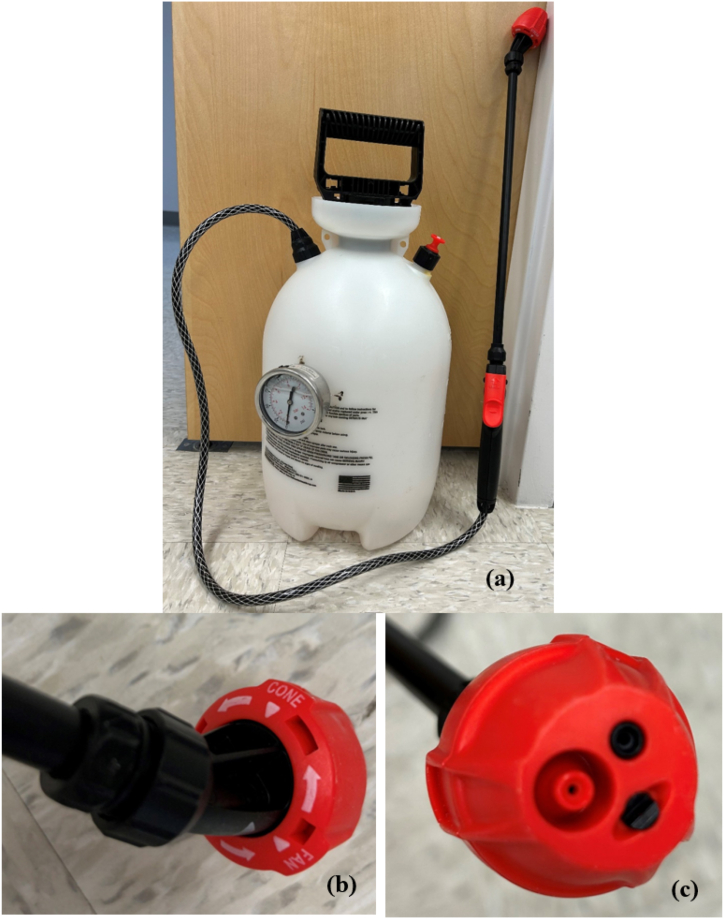
Sprayer and nozzles used in the experiment. (a) Sprayer, (b) knob for changing nozzle, and (c) nozzles.

In the laboratory, the experiments were performed with a simple backpack sprayer with cone and flat fan nozzles (Fig. 1). The spray fluids were pressurized to 131, 172, and 200 kPa. To maintain consistent pressure in each trial, the tank was fitted with a pressure gauge and the pressure was controlled by the handle used to pressurize the tank. Three different adjuvant concentrations (weight of adjuvant/volume of water), namely, 1.25, 2.5, and 5 g/L (estimated by trial and error) for SA and 75, 150, and 300 μg/L for PAM were used in the experiments. In all the experiments, the insecticide surrogate (triphenyl phosphate) dose was kept the same at 313 μL/L water. The entire laboratory experiments were carried out in four sets: (i) water only, (ii) water and adjuvant, (iii) water and pesticide surrogate, and (iv) water, pesticide surrogate and adjuvant. Each set contained 18 treatment combinations (2 × 3 x 3) to cover the ranges in spray pressure, adjuvant dose, and the two different nozzles.

### 2.4 Estimation of spray droplet spectrum and velocity

To analyze the spray droplet spectrum and spray velocity, a VisiSizer P15 (Oxford Lasers, UK) image analysis system was used. This image analysis system uses a short, double light pulse to illuminate a plane of spray that is photographed such that droplets show up as dark spots against a bright background. A digital camera is used to capture the snapshots of the spray particles. The instrument has a sealed housing that is resistant to chemicals. The instrument can be attached to a desktop/laptop computer and proprietary VisiSizer software (which comes with the instrument) uses image shape thresholds to identify the in-focus droplets in the image and determine their sizes and velocities. Three different magnification settings are available to target a specific range of spray droplets. Magnifications of 2, 1, and 0.56 correspond to droplet diameters of 10 μm−1000 μm, 21 μm−2039 μm, and 41 μm−3543 μm, respectively [40]. VisiSizer P15 can be used as a standalone instrument to analyze pesticide droplet spectra in the laboratory or placed in the field to analyze how the sprayed pesticide is deposited on the surface of leaves or drifts elsewhere. The instrument can also provide close-up images of the spray formation and break-up process. The parameters displayed by the instrument are average diameter (number, area and volume), Sauter mean diameter (D_32_), 10 %, 50 % and 90 % percentile volumes (DV_10_, DV_50_, and DV_90_), standard deviation, relative span and all the curves related to the parameters describing the droplet spectrum. The maximum spray velocity is approximately 10 m/s (for 50 μm diameter particles). Up to 15,000 particles/second can be analyzed using this instrument (http://aams-salvarani.com/en/products/oxford-laser-p15; https://www.oxfordlasers.com/laser-imaging/visisize-p15) [[41], [42]]. The droplet spectrum measurement system used in this study is consistent with the standards prescribed by the American Society of Agricultural and Biological Engineers (ASABE S572.3) [22].

### 2.5 Analysis of data

Although the image analysis instrument provides several parameters, one of the most important ones for characterizing drift is the driftable proportion of spray volume (droplets with diameter less than 150 μm [[43], [44], [45]]). The spray velocity is also important because the spray particles traveling more slowly to hit the target are more likely to drift away from the target. When added to the pesticide tank mix, it is expected for the adjuvant to send the spray particles to the target quicker. The drift reduction potential of SA is analyzed by (i) dose, (ii) spray pressure and (ii) nozzle type. In addition, the drift reduction potential of SA is compared to PAM as a reference.

#### 2.5.1 Particle diameter distribution regression model

A Bayesian hierarchical mixed-effects distributional regression model was fit to the particle diameter data. The midpoint of the upper and lower bounds of each diameter bin in the image analyzer output (raw data) was calculated and assumed to represent the diameter of each particle. We assumed a lognormal response distribution. Both the mean (μ) and standard deviation (σ) parameters of the lognormal distribution were fit with a combination of fixed and random effects. All fixed effects were categorical: adjuvant (three levels: none, SA, and PAM), adjuvant dose (four levels: none, which was only present in the no-adjuvant treatment; low, medium, and high, which were only present in the two adjuvant treatments), nozzle type (two levels: cone and fan), pesticide (two levels: absent and present), and pressure (three levels: 131, 172, and 200 kPa). Second-order and third-order interaction terms were included. Normal prior distributions with mean 0 and standard deviation 1 were assigned to all fixed effect parameters. Random intercepts were fit to each experimental run. The joint posterior distribution of parameters was sampled using Hamiltonian Monte Carlo, with 4 Markov chains each running for 4000 warmup iterations and 2000 sampling iterations. Convergence was assessed by ensuring that the potential scale reduction factor was ≤1.01 for all parameters [46].

#### 2.5.2 Estimation and comparison of treatment means

We estimated the posterior distributions of the marginal means of the μ and σ parameters for each adjuvant at each dose level, both averaged across the nozzle, pesticide, and pressure treatments, and separately within each of those treatments. We also estimated the marginal means of the parameters for SA and PAM averaged across the low, medium and high dose levels, again both averaged across the other treatments and separately within each treatment. We used these values to calculate the posterior estimated marginal means of Q_10_, Q_50_, and Q_90_ (corresponding to DV_10_, DV_50_ and DV_90_) and the proportion of droplets with diameter <150 μm for all abovementioned treatment combinations. We calculated pairwise contrasts between each pair of adjuvant treatments by taking the ratio of their Q_10_, Q_50_, Q_90_, and proportion <150 μm values. For all posterior estimated marginal means and ratios, the median of the distribution is presented as a point estimate, and quantile credible intervals (QCI; 50 %, 80 %, and 95 %) are presented as a measure of estimation uncertainty. The posterior probabilities of direction (p_d_) that each ratio was greater or less than 1 (no difference between adjuvants) were also calculated [47].

Statistical analysis was done using R v4.3.1 [48], Stan v2.33.1 [49], and R packages brms v2.20.5 [50], cmdstanr v0.6.1 [51], and emmeans v1.8.9 [52]. Data and code to reproduce analyses will be deposited in Ag Data Commons.

#### 2.5.3 Field testing of the drift reduction potential of sodium alginate

Three different field experiments were performed to test the drift reduction potential of SA. In all three experiments, SA at 1.25 g/L concentration in water was tested. In the first experiment (CA1 of Table 2), 1.25 g/L of SA was tested against water alone. In each spray experiment, 11 trials were carried out. The VisiSizer P15 image analyzer was used to estimate the size and velocity of droplets forming the spray. In the second experiment (CA2), fluometuron (commercial formulation: Cotoron) with and without SA was applied at 3.5 L/ha. Five trials each were carried out for fluometuron and fluometuron with SA. For logistic reasons, the third experiment was split into two parts with chemical application on two different days. The first part (CA3) involved the application of glufosinate-ammonium (commercial formulation: Liberty) alone at 2.3 L/ha (four trials), and the second part (CA4) involved the application of glufosinate-ammonium along with SA (four trials). For all the field experiments, a Kubota RTV vehicle fitted with a 170 L spray tank (Fig. S1) was driven at 3.3 km/h. The sprayer boom had seven AIXR11002VP (Teejet) air induction flat fan spray nozzles spaced at 51 cm. The chemical was sprayed from 60 cm height from the target at 276 kPa pressure to form a swath of 4.6 m. The average single nozzle flow rate was 39.1 L/h (274 L/h of total flow rate from all the 7 nozzles). For the second and third field application experiments, water sensitive papers placed at 25.4 cm distance were used to obtain the spray droplet stains, which were then analyzed by AccuStain software (University of Illinois, Champaign-Urbana, IL) using the scanned image of the water sensitive paper with droplet stains. Critical weather parameters affecting off-target pesticide drift, such as wind speed, wind direction, air temperature and relative humidity, were observed using professional grade LiCor weather sensors (Table 2).

**Table 2:** Weather parameters recorded during field experiments in 2023.

Date and time	Chemical application	Wind speed (m/s)	Wind direction (deg from N)	Air temp. (°C)	Relative humidity (%)
Mar 13 3:30 to 4:30 p.m. May 26 9:00 to10:30 a.m. Jun 21 11:51 to 1:44 p.m. Jun 22 1:07 to 1:32 p.m.	CA 1 CA 2 CA 3 CA 4	4.2 to 4.3 1.3 to 2.2 1.8 to 2.2 1.6 to 1.9	303.0 to 305.2 16.2 to 58.8 20 to 25 20 to 25	10.9 to 11.0 23.9 to 27.0 32.5 to 36.5 34.0 to 35.5	44.7 to 47.7 54.4 to 64.7 42.0 to 50.0 42.0 to 48.0

Note.

CA 1: Water only, water and SA application.

CA 2: Fluometuron alone, Fluometuron and SA.

CA 3: Glufosinate-ammonium application.

CA 4: Glufosinate-ammonium with SA.

### 2.6 Laboratory testing of the drift reduction potential of sodium alginate for the specific nozzle used in the field experiment

**Fig. 2:**
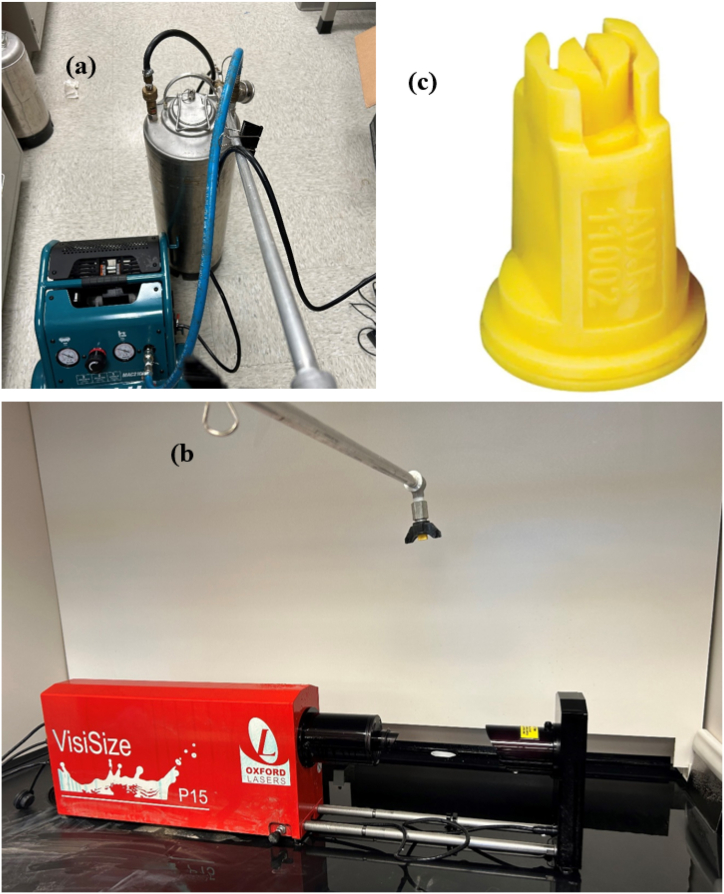
Experimental setup used for testing nozzle AIXR 11002 (a) spray setup (b) VisiSize P15 image analyzer (c) nozzle.

We designed a spray system to test the drift reduction potential of SA. The spray system consists of a Makita air compressor (Mac210Q with 1 HP motor and a 7.5 L tank), a liquid storage tank (metal canister with a pressure rating of 896 kPa), a metal spray boom with an inbuilt pressure gauge (can read until 413 kPa (60 PSI)) (Fig. 2). The hose connecting the compressor and liquid storage tank can withstand a pressure of 2000 kPa. The spray system has provisions to change the nozzle. The entire laboratory experiments were carried out in two sets: (i) water only as control, (ii) water and adjuvant as treatment. The control consisted of spraying water at seven different pressures from 138 kPa (20 PSI) to 345 kPa (50 PSI) in steps of 34.5 kPa (5 PSI). For each trial of the experiment, the desired pressure was regulated by the lever available in the air compressor in combination with the inbuilt pressure gauge available in the spray boom. In each experiment (control/treatment), 12 trials were taken with 4 trials each at 14 cm, 27 cm, and 37 cm height from the point of image analyzer observation to the point of fluid discharge from the nozzle tip. This experimental setup complies with the standards prescribed by the American Society of Agricultural and Biological Engineers (ASABE S572.3) [22]. The only deviation to the standard is the small reduction in height which was provided to avoid frequent fouling of the lens used in the image analyzer by spray droplets. Three different adjuvant concentrations namely, 1.25, 2.5, and 5 g/L were tested using the experimental setup described above.

Statistical analysis was carried out by fitting linear models to the DV_10_, D_32_, DV_50_, DV_90_, and proportion of droplets <150 μm from the laboratory trials testing different concentrations of sodium alginate. The data were analyzed as a completely randomized design. Residuals were approximately normally distributed and homoscedastic. Analysis of variance (ANOVA) and Tukey post hoc test was used to assess significance of pairwise differences between means.

## 3 Results and discussion

### 3.1 Physicochemical characteristics of the adjuvants

For both adjuvants, the density of the adjuvant solution did not change with respect to dose. However, the pH progressively increased with higher dose for both adjuvant solutions. For SA, pH increased by 0.87 %, and 1.9 % for low-medium and low-high doses. The similar values for PAM are 2.74 % and 3.26 % respectively. It should be noted that with reference to water, PAM solution is relatively more acidic than SA solution (Table 1). In case of viscosity, SA exhibited dramatic increases with dose. Addition of SA to water increased the viscosity by 64.3 %, 117.0 % and 221.3 % respectively for low, medium and high doses. For PAM, the viscosity increased slightly from a low to medium dose. However, for unknown reasons, the viscosity decreased from medium to high doses, collectively suggesting that PAM did not significantly change the viscosity. Viscosity increases of PAM with respect to water are 13.2 %, 24.8 % and 12.4 % respectively for low, medium and high doses. Appreciable reductions in surface tensions were seen for PAM with increasing doses (with respect to water 3.5 %, 8.3 % and 13.3 % reductions for low, medium, and high doses respectively), whereas SA did not show reductions in surface tension (Table 1). The addition of simple ingredients such as surfactant is adequate to decrease the surface tension of SA solution. It should be noted that SA, which is only a thickener, was used as a potential less toxic/alternate pesticide adjuvant in the study. However, PAM has been available to industry as a proven pesticide adjuvant for several decades.

### 3.2 Drift reduction results by dose

**Fig. 3:**
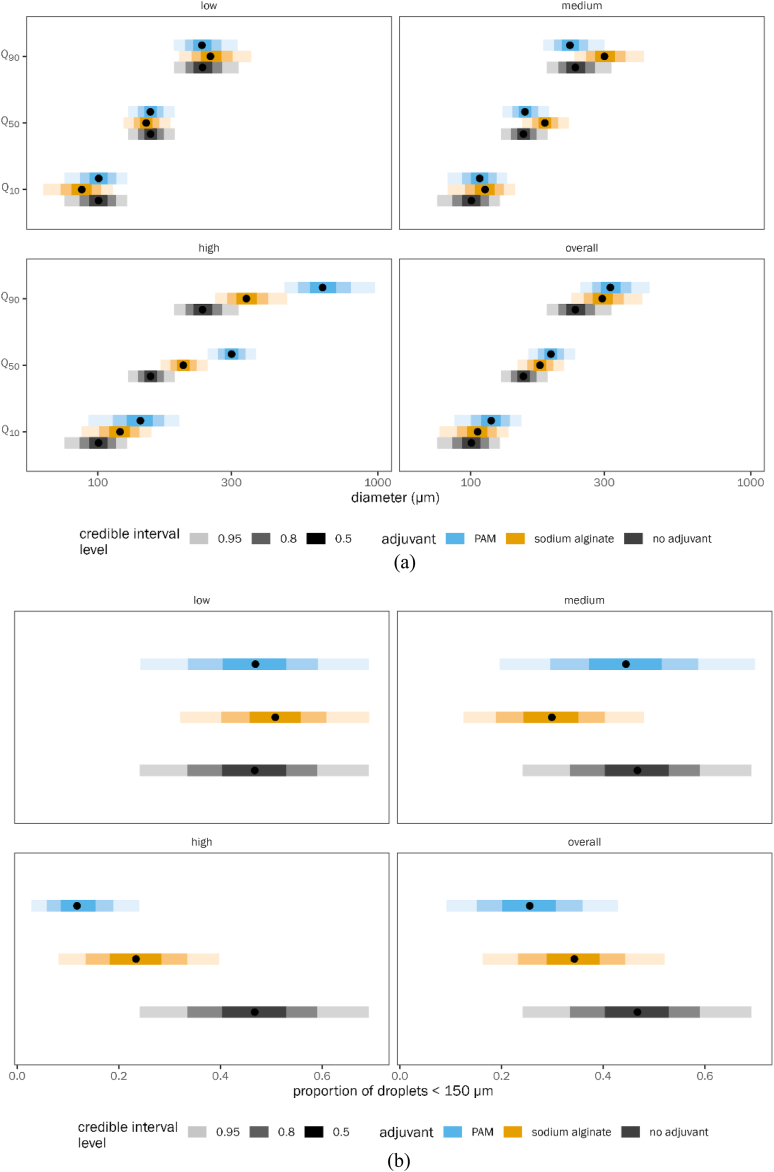
Effect of adjuvant dose on drift reduction potential of spray mix (a) droplet size and (b) proportion of droplets less than 150 μm. PAM is polyacrylamide. Points represent medians of the posterior distribution of the estimated marginal mean for each group, with progressively lighter-shaded error bars indicating the 50 %, 80 %, and 95 % quantile credible interval around the point estimate. Each panel shows a different adjuvant dose level, as well as marginal means averaged across all dose levels. (Note: Quantiles Q_10_, Q_50_ and Q_90_ correspond to droplet spectrum parameters DV_10_, DV_50_ and DV_90_ respectively).

Fig. 3a is shown with posterior estimated means of Q_10_, Q_50_, and Q_90_ of droplet diameter, with credible interval error bars. These are shown separately by dose for PAM and SA and averaged across doses. The same “no adjuvant” value is repeated across all the panels for comparison with the low, medium, and high doses. The table of estimated marginal means (Table S1 supplementary materials) shows posterior means along with quantiles along with 95 % quantile credible intervals for Q_10_, Q_50_, Q_90_, and proportion <150 μm.

Fig. 3b is shown with posterior estimated means of the proportion of droplets <150 μm, with credible interval error bars. These are shown in the same way as the quantile figures described above.

The contrasts shown in Table 3 are in the form of ratios. A ratio of 1 indicates no difference. The 95 % credible interval of the ratio is also given. The posterior probability that the ratio is different than 1 is also provided alongside. For example, in column 5 and row 11 of Table 3, we see that the contrast between SA at medium dose and no adjuvant is 0.64 for a proportion of droplets <150 μm, with a 95 % credible interval of 0.45–0.77. This means that we estimate that SA at a medium dose reduces the proportion of droplets <150 μm to 0.64 of what it is with no adjuvant at all, and there is 95 % probability that this value is between 0.45 and 0.77. Additionally, the posterior probability of direction (p_d_) that this value is less than 1 is > 0.999 (see Table 4).

**Table 3:** Results of contrasts summarized by adjuvant dose (Note: Quantiles Q_10_, Q_50_ and Q_90_ correspond to droplet spectrum parameters DV_10_, DV_50_ and DV_90_ respectively).

	SA/PAM	SA/none	PAM/none	SA/PAM	SA/none	PAM/none	SA/PAM	SA/none	PAM/none	SA/PAM
Dose	Low	Low	Low	Medium	Medium	Medium	High	High	High	All
Pesticide	Included	Included	Included	Included	Included	Included	Included	Included	Included	Included
Nozzle	Both	Both	Both	Both	Both	Both	Both	Both	Both	Both
Pressure (kPa)	All	All	All	All	All	All	All	All	All	All
Q_10_	0.87 (0.82–0.90)	0.87 (0.82–0.91)	1.00 (0.99–1.02)	1.04 (0.98–1.09)	1.12 (1.08–1.15)	1.07 (1.05–1.11)	0.85 (0.76–0.97)	1.19 (1.12–1.26)	1.41 (1.18–1.60)	0.89 (0.87–0.92)
Post. Prob Q_10_	>0.999	>0.999	0.610	0.937	>0.999	>0.999	0.985	>0.999	0.996	>0.999
Q_50_	0.97 (0.95–0.98)	0.96 (0.95–0.98)	1.00 (0.99–1.01)	1.18 (1.16–1.20)	1.19 (1.17–1.21)	1.01 (1.00–1.03)	0.67 (0.63–0.72)	1.31 (1.26–1.35)	1.95 (1.83–2.06)	0.91 (0.89–0.94)
Post. Prob Q_50_	>0.999	>0.999	0.576	>0.999	>0.999	0.966	>0.999	>0.999	>0.999	>0.999
Q_90_	1.07 (1.03–1.13)	1.07 (1.03–1.13)	1.00 (0.98–1.01)	1.33 (1.27–1.42)	1.27 (1.23–1.32)	0.96 (0.93–0.98)	0.53 (0.46–0.59)	1.43 (1.37–1.53)	2.69 (2.37–3.21)	0.94 (0.91–0.96)
Post. Prob Q_90_	>0.999	0.999	0.724	>0.999	>0.999	>0.999	>0.999	>0.999	>0.999	>0.999
Prop<150 μm	1.08 (0.99–1.33)	1.09 (0.99–1.34)	1.00 (0.96–1.04)	0.68 (0.51–0.80)	0.64 (0.45–0.77)	0.95 (0.80–1.02)	1.95 (1.36–3.86)	0.50 (0.28–0.67)	0.26 (0.08–0.46)	1.34 (1.16–1.85)
Post. Prob	>0.960	0.967	0.558	>0.999	>0.999	0.900	>0.999	>0.999	>0.999	>0.999

From Fig. 3a and b and from the table of contrasts (Table 3), we learn that at low doses, PAM has a slightly larger Q_10_ diameter than SA (118.4 μm with 95 % QCI [87.2, 115.9] versus 105.9 μm with 95 % QCI [77.3, 136.5]) and a slightly smaller Q_90_ diameter than SA. However, the differences are small. At a medium dose, SA is more effective at increasing the particle size of Q_50_ (1.18 times more effective than PAM; QCI [1.16, 1.20]) and Q_90_ (1.33 times more effective; QCI [1.27, 1.42]). At high doses, it appears that PAM is much more effective at increasing the particle size, especially at Q_50_ (SA 0.67 as effective as PAM; QCI [0.63, 0.72]) and Q_90_ (SA 0.53 times as effective; QCI [0.46, 0.59]). Thus, the relative effectiveness of the two adjuvants is dose-dependent (similar at low doses, SA is slightly better at medium doses, and PAM is better at high doses). Averaging the marginal means across the doses, there is no evidence for overall difference between PAM and SA (columns 3 and 4 of Table 3) because the conditions where PAM is higher “cancel out” the conditions where SA is higher.

In terms of reducing the proportion of driftable droplets (<150 μm), both PAM and SA at low doses did not make any improvements, with no evidence for difference from the control (control proportion 0.467, QCI [0.241, 0.692]; PAM 0.469, QCI [0.242, 0.692]; SA 0.508 [0.320, 0.692]) (Table S1). SA at a medium dose (0.299, QCI [0.125, 0.480]) performed better than PAM (0.445, QCI [0.196, 0.699]) in minimizing driftable droplets. However, at high doses, PAM (0.118, QCI [0.028, 0.240]) did a better job than SA (0.234, QCI [0.081, 0.398]). In summary, when added to the tank mix at medium and high doses, both PAM and SA were able to reduce the proportion of driftable droplets. SA performed as well as PAM. However, the drift reduction abilities of PAM appear to be better than those of SA at the highest dose.

### 3.3 Drift reduction results by pressure

**Fig. 4:**
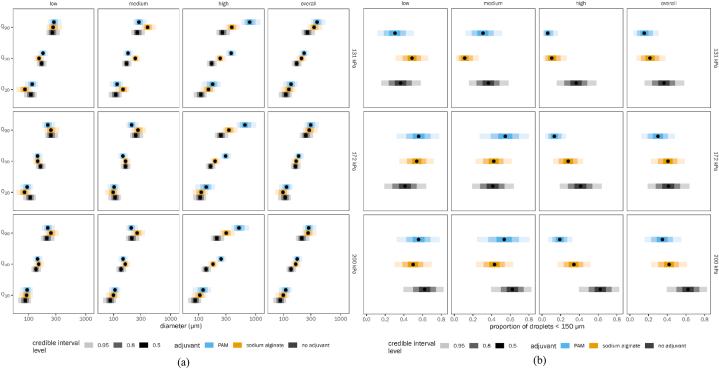
Effect of spraying pressure on drift reduction potential of spray mix: (a) droplet size and (b) proportion less than 150 μm. Each panel shows a different combination of adjuvant dose level and spraying pressure, as well as marginal means for each pressure averaged across all adjuvant dose levels. See Fig. 3 legend for explanation of points and error bars. (Note: Quantiles Q_10_, Q_50_ and Q_90_ correspond to droplet spectrum parameters DV_10_, DV_50_ and DV_90_ respectively).

Irrespective of pressure, at low dose, both PAM (no detectable increase in Q_50_ or Q_90_ relative to control) and SA (Q_50_ reduced by a factor of 0.96, QCI [0.95, 0.98]; Q_90_ increased by a factor of 1.07, QCI [1.03, 1.13] relative to control) are ineffective in increasing droplet size (Fig. 4a and Table S2 And Table 4) and reducing the proportion of driftable droplets (Fig. 4b and Table S2 And Table 4). At a medium dose and at both low and high pressures, SA performed better than PAM in increasing droplet size and controlling drift (SA: proportion driftable droplets reduced relative to control by a factor of 0.31, QCI [0.1, 0.53] at low pressure, 0.69 [0.53, 0.81] at high pressure; PAM: proportion driftable droplets reduced relative to control by a factor of 0.84 [0.69, 0.93] at low pressure, 0.86 [0.6, 0.99] at high pressure). However, the same trend cannot be seen for medium pressure. For all pressures, at a high dose of adjuvant, both PAM and SA controlled drift very well, with PAM performing better than SA (1.7, QCI [0.81, 4.25] times less reduction in driftable droplets for SA relative to PAM at low pressure; 1.21 [1.07, 1.54] times less at high pressure). The overall results (considering all the doses of adjuvants) showed that both PAM and SA increased the droplet size and minimized the proportion of driftable droplets. Additionally, we noticed a progressive decrease in drift reduction potential as pressure increased from low to high for both adjuvants. In summary, based on pressure, we could also see a better performance by PAM than SA.

**Table 4:** Results of contrasts summarized by spray pressure (Note: Quantiles Q_10_, Q_50_ and Q_90_ correspond to droplet spectrum parameters DV_10_, DV_50_ and DV_90_ respectively).

	SA/PAM	SA/none	PAM/none	SA/PAM	SA/none	PAM/none	SA/PAM	SA/none	PAM/none	SA/PAM
Dose	All	All	All	All	All	All	All	All	All	All
Pesticide	Included	Included	Included	Included	Included	Included	Included	Included	Included	Included
Nozzle	Both	Both	Both	Both	Both	Both	Both	Both	Both	Both
Pressure (kPa)	131	131	131	172	172	172	200	200	200	All
Q_10_	0.92 (0.87–0.97)	1.36 (1.22–1.49)	1.60 (1.31–1.86)	0.86 (0.83–0.90)	1.04 (0.94–1.13)	1.28 (1.03–1.50)	0.90 (0.87–0.94)	1.20 (1.11–1.27)	1.37 (1.15–1.56)	0.89 (0.87–0.92)
Post. Prob Q_10_	>0.999	>0.999	0.999	>0.999	0.798	0.983	0.985	>0.999	0.997	>0.999
Q_50_	0.90 (0.86–0.94)	1.41 (1.31–1.52)	2.19 (1.99–2.42)	0.90 (0.87–0.93)	1.20 (1.13–1.27)	1.84 (1.68–2.01)	0.94 (0.91–0.97)	1.32 (1.27–1.38)	1.83 (1.70–1.96)	0.91 (0.89–0.94)
Post. Prob Q_50_	>0.999	>0.999	0.576	>0.999	>0.999	>0.999	>0.999	>0.999	>0.999	>0.999
Q_90_	0.89 (0.84–0.93)	1.46 (1.35–1.61)	3.00 (2.57–3.73)	0.94 (0.90–0.99)	1.39 (1.28–1.53)	2.64 (2.25–3.29)	0.98 (0.94–1.02)	1.46 (1.37–1.58)	2.44 (2.16–2.88)	0.94 (0.91–0.96)
Post. Prob Q_90_	>0.999	0.999	>0.999	0.992	>0.999	>0.999	0.886	>0.999	>0.999	>0.999
Prop<150 μm	1.38 (1.14–2.06)	0.29 (0.07–0.54)	0.17 (0.03–0.39)	1.36 (1.15–1.94)	0.69 (0.48–0.85)	0.32 (0.12–0.55)	1.21 (1.07–1.54)	0.55 (0.35–0.70)	0.31 (0.13–0.50)	1.34 (1.16–1.85)
Post. Prob	>0.960	0.967	>0.999	>0.999	0.998	>0.999	>0.999	>0.999	>0.999	>0.999

### 3.4 Drift reduction results by nozzle

**Fig. 5:**
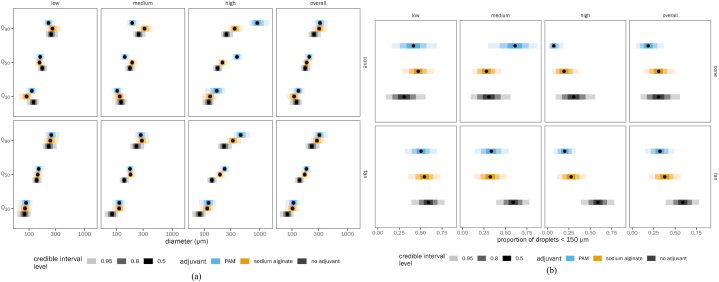
Effect of nozzle type on drift reduction potential of spray mix: (a) droplet size and (b) proportion less than 150 μm. Each panel shows a different combination of adjuvant dose level and nozzle type, as well as marginal means for each nozzle type averaged across all adjuvant dose levels. See Fig. 3 legend for explanation of points and error bars. (Note: Quantiles Q_10_, Q_50_ and Q_90_ correspond to droplet spectrum parameters DV_10_, DV_50_ and DV_90_ respectively).

For the cone nozzle, at a low dose, both PAM and SA were ineffective in increasing droplet size (Fig. 5a and Tables S3 and 5) and reducing the proportion of driftable droplets (Fig. 5b and Tables S3 and 5). At a medium dose, SA performed better than PAM in increasing droplet size (SA/PAM Q_90_ ratio 1.67, QCI [1.55, 1.86]) and controlling drift (SA/PAM proportion driftable droplets ratio 0.46, QCI [0.27, 0.63]). At a high dose of adjuvant with a cone nozzle, PAM performed better than SA (SA/PAM Q_90_ ratio 0.39 [0.31, 0.47]; proportion driftable droplets ratio 2.56 [1.42, 7.68]). For flat fan nozzles as well, both adjuvants are not effective in controlling drift at low doses. For the medium dose with flat fan nozzle, both adjuvants performed similarly. However, at high doses, PAM performed better than SA (SA/PAM Q_90_ ratio 0.72 [0.65, 0.78]; proportion driftable droplets ratio 1.36 [1.10, 1.90]). Overall, both PAM and SA increased the droplet size and minimized the proportion of driftable droplets. The effect of nozzle type on performance did not differ markedly between PAM and SA. The drift reduction caused by the flat fan nozzle was greater than that of the cone nozzle. Additionally, most of the drift reductions tended to occur between Q_50_ and Q_90_ droplet sizes.

**Table 5:** Results of contrast summarized by nozzle type (Note: Quantiles Q_10_, Q_50_ and Q_90_ correspond to droplet spectrum parameters DV_10_, DV_50_ and DV_90_ respectively).

Item	Sodium alginate/polyacrylamide	Sodium alginate/polyacrylamide
Dose	All	All
Pesticide	Included	Included
Nozzle	Cone	Fan
Pressure (kPa)	All	All
Q_10_	0.83 (0.79–0.88)	0.96 (0.94–0.99)
Posterior Probability Q_10_	0.993	0.993
Q_50_	0.90 (0.86–0.93)	0.93 (0.91–0.95)
Posterior Probability Q_50_	>0.999	>0.999
Q_90_	0.96 (0.92–1.02)	0.90 (0.88–0.93)
Posterior Probability Q_90_	0.903	>0.999
Prop<150 μm	1.65 (1.24–3.13)	1.17 (1.09–1.35)
Posterior Probability	>0.999	>0.999

### 3.5 Drift reduction results by pesticide

**Fig. 6:**
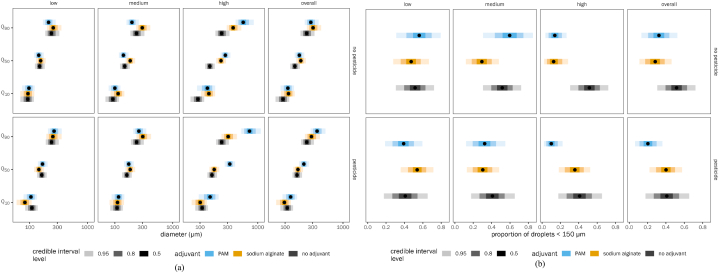
Effect of pesticide on the drift reduction potential: (a) droplet size and (b) proportion less than 150 μm. Each panel shows a different combination of adjuvant dose level and presence of pesticide, as well as marginal means with and without pesticide averaged across all adjuvant dose levels. See Fig. 3 legend for explanation of points and error bars. (Note: Quantiles Q_10_, Q_50_ and Q_90_ correspond to droplet spectrum parameters DV_10_, DV_50_ and DV_90_ respectively).

Irrespective of the presence/absence of pesticide, at a low dose, both PAM and SA were ineffective in increasing droplet size (Fig. 6a and Tables S4 and 6) and reducing the proportion of driftable droplets (Fig. 6b and Tables S4 and 6). At medium and high doses, both adjuvants minimized drift by increasing droplet size and minimizing the proportion of driftable droplets. In the presence of pesticides, SA performed marginally better than PAM at reducing proportion of driftable droplets at medium dose only (ratio 0.94, QCI [0.83, 1.14]). However, PAM had a better performance in minimizing drift than SA at higher doses (Fig. 6a and Tables S4 and 6; ratio 3.50, QCI [1.96, 11.57]). In the absence of pesticide, SA performed better at medium doses (ratio 0.50, QCI [0.31, 0.65]). In either case, drift reductions tended to occur between Q_50_ and Q_90_ droplet sizes for both adjuvants.

**Table 6:** Results of contrast summarized by presence of pesticide (Note: Quantiles Q_10_, Q_50_ and Q_90_ correspond to droplet spectrum parameters DV_10_, DV_50_ and DV_90_ respectively).

	Sodium alginate/Polyacrylamide	Sodium alginate/Polyacrylamide
Dose	All	All
Pesticide	No	Included
Nozzle	All	All
Pressure (kPa)	All	All
Q_10_	1.02 (0.99–1.06)	0.78 (0.75–0.81)
Post. Prob Q_10_	0.904	>0.999
Q_50_	1.06 (1.03–1.09)	0.79 (0.77–0.82)
Post. Prob Q_50_	>0.999	>0.999
Q_90_	1.09 (1.06–1.14)	0.80 (0.77–0.83)
Post. Prob Q_90_	>0.999	>0.999
Prop<150 μm	0.87 (0.75–0.96)	1.97 (1.44–3.73)
Post. Prob	0.996	>0.999

We conclude based on our observations of droplet diameter in laboratory experiments that the proposed algae-based polymer adjuvant (SA) is able to reduce off-target drift when compared to the no adjuvant scenario and is almost as good as the commercially used adjuvant PAM. This pattern was largely consistent across dose, nozzle type, and pressure. In the presence of pesticide, PAM shows appreciable reductions in drift at medium and high doses, whereas SA shows drift reductions at medium dose only.

### 3.6 Drift reduction results by droplet velocity

This section focuses on the droplet velocity (which depends on the size of droplets) in relation to changes in other parameters. The droplet velocity analysis was performed in detail similar to what was described in the previous section. However, for brevity, only key results are summarized in this section.

**Fig. 7:**
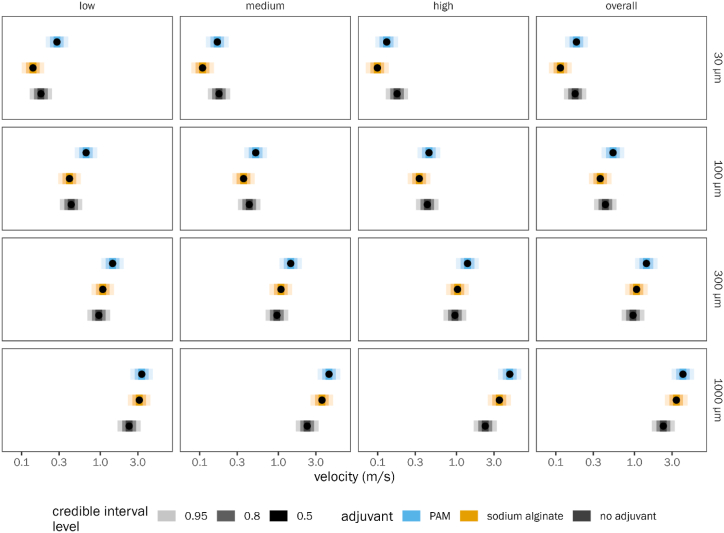
Effect of adjuvant material on droplet velocity across a range of particle sizes. Each panel shows a different combination of adjuvant dose level and particle size (30 μm, 100 μm, 300 μm, and 1000 μm), as well as marginal means averaged across all three adjuvant dose levels. See Fig. 3 legend for explanation of points and error bars.

The effect of adjuvant was different at different particle sizes: for small particles, SA slowed down the particles more than PAM (SA/PAM velocity ratio 0.71, QCI [0.70, 0.72] at 30 μm). For large particles (>300 μm), both SA (ratio vs. control 1.51, QCI [1.40, 1.62]) and PAM (ratio vs. control 2.05, QCI [1.93, 2.17]) caused the particles to move faster than controls without adjuvant, but this effect was somewhat greater for PAM. Averaged across all treatments and all diameters, PAM had a faster droplet velocity than SA. Size-dependent changes in droplet velocity were more prominent than dose-dependent changes in droplet velocity (Fig. 7).

The difference between droplet velocity between flat fan and cone nozzle type was not noticeable for SA, but it was larger for PAM. For many particle sizes, the droplet velocity when PAM was added was higher for the flat fan nozzle than for the cone nozzle (Figure S2 eg. 300 μm velocity 1.63 m/s, QCI [1.17, 2.26] for flat fan nozzle; 1.23 [0.88, 1.71 for cone nozzle)). For smaller particles (<300 μm), there were little to noticeable differences in droplet velocities between the nozzle types.

The effect of pesticide on droplet velocity was relatively minor for all particle sizes for both adjuvants, with only minor and inconsistent effects at the smallest sizes (Fig. S3). The effect of pressure on droplet velocity was relatively small for most particle sizes for SA. However, for the smallest particles, there was some interaction between pressure and velocity for both adjuvants (Fig. S4). Specifically, the slowing effect of both adjuvants was greater at low pressures for small particles (30 μm) but did not depend on pressure for larger particles.

Based on the droplet velocity results from all the experiments, it can be interpreted that the proposed algae-based polymer adjuvant (SA) can reduce off-target drift by marginally increasing the droplet velocities especially of large particles. The drift reduction results exhibited by SA are less in terms of droplet velocity than in terms of droplet diameter, although increases in both parameters are desirable to reduce off-target drift. PAM, which is a proven drift reducing agent, shows appreciable increases in both droplet size and velocity. One important point to note is that SA at a medium dose performs better relative to PAM than at low and high doses.

### 3.7 Drift reduction results from field experiments

The droplet spectrum results from VisiSizer P15 are presented in Table 7 for water with SA and water alone. In the first experiment (CA1), the air temperature, wind speed and direction were relatively stable throughout the experiment. The only parameter that varied during the experiment was the relative humidity (Table 2). The average results of 11 trials in each setup suggest that the addition of SA produces larger droplets that fall faster than the droplets of water.

**Table 7:** Field experiment results observed by the P15 Oxford Laser image analyzer.

Tank mix	Droplet spectrum and droplet velocity			
	DV_10_ (μm)	DV_50_ (μm)	DV_90_ (μm)	Velocity (m/s)
Water^a^ Water and sodium alginate^a^	270.4 276.5	432.1 434.0	639.3 699.9	3.03 3.11

In the second experiment, droplet stain data collected on water-sensitive cards and analyzed using AccuStain software were compared for fluometuron with adjuvant solution and fluometuron alone (Table 8). The wind speed and wind direction were stable throughout the experiment. However, the air temperature and relative humidity varied to some extent. The results presented in Table 8 suggest that fluometuron with SA produced better on-target coverage and larger droplets (DV_50_ and DV_90_) than fluometuron alone. Although the DV_10_ result for the pesticide solution with SA was smaller than that for pesticide alone, it did not affect the overall on-target results that indirectly show reductions in drift. Furthermore, fluometuron with SA produced less off-target drift than fluometuron alone. It is exhibited by the reduction in spray coverage (less is desired for off-target drift) and reduction in droplet number (per unit area) for pesticide with SA than water and pesticide alone.

**Table 8:** Field experiment data collected on water sensitive papers analyzed by AccuStain software.

Tank mix	On target parameters				Off target parameters	
	% coverage	DV_10_	DV_50_	DV_90_	Drops/cm^2^	% coverage
Fluometuron^a^ Fluometuron with sodium alginate^a^ Glufosinate-ammonium^b^ Glufosinate-ammonium with sodium alginate^b^	28.5 29.7 13.8 16.9	273.7 258.4 222.1 270.2	460.4 466.9 356.0 451.1	625.9 657.0 524.1 635.2	34.0 17.1 11.0 18.5	0.34 0.16 0.22 0.23

In the third field experiment, glufosinate-ammonium application with and without adjuvants was compared. Although the off-target spray coverage results show similar values for the trials with and without adjuvant, the on-target results clearly show increased spray coverage and appreciably larger droplets throughout the droplet spectrum for pesticide spray with SA than pesticide alone, which suggests a possible reduction in spray drift using SA. Throughout the experiment, there was little variation in air temperature and relative humidity. The wind direction remained stable. However, the wind speed decreased during the spray experiment for pesticides with adjuvants compared with pesticides alone (Table 2), which could have influenced some of the drift reduction results indicated by the larger droplet size. In addition, the results from all three field experiments collectively indicate reductions in off-target pesticide drift when SA is used as an adjuvant.

**Fig. 8:**
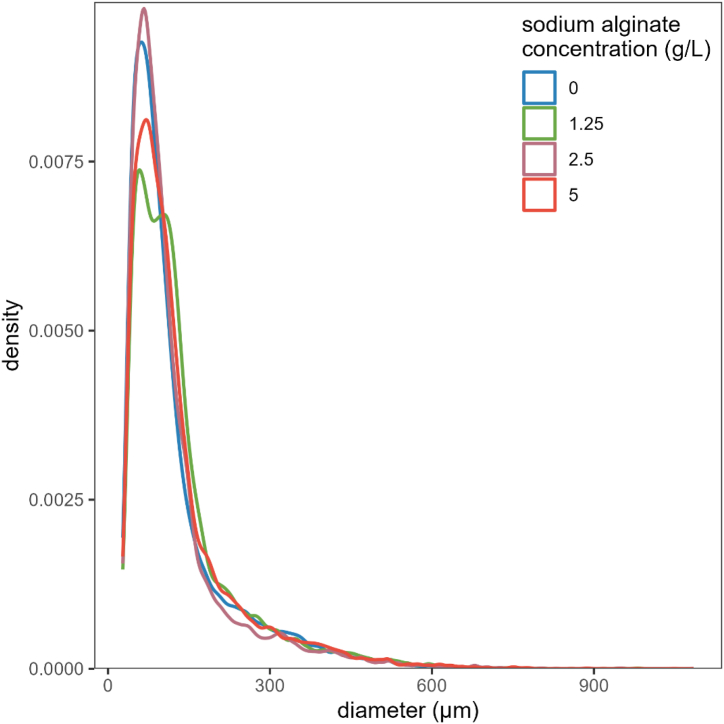
Density plot of droplet diameters observed by the P15 Oxford Laser image analyzer for air induction flat fan nozzle AIXR 11002.

**Fig. 9:**
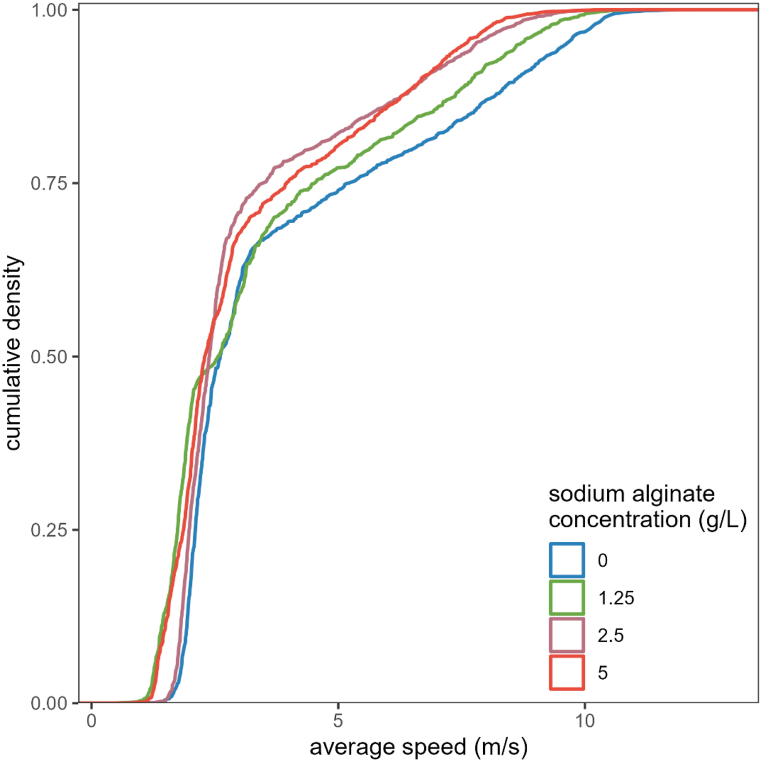
Cumulative density plot of droplet velocity observed by the P15 Oxford Laser image analyzer for air induction flat fan nozzle AIXR 11002.

The statistical analysis of results for the AIXR 11002 nozzle indicates that mean of DV_10_, and DV_50_ are significantly greater for the sodium alginate concentrations relative to the control (Table 9, Fig. 8), but for DV_90_ there was no significant difference for any levels. The proportion of droplets with diameter less than 150 μm has significant differences between the concentrations of sodium alginate but not between adjuvant and control (Table 9). There is a decrease in the velocity for water and adjuvant solution droplets when compared to control (Fig. 9). This is true for all the concentrations. However, the effect is less pronounced for SA at 1.25 g/L. This agrees well with the trends in density of SA solutions when compared to water. An interesting thing to note is SA at 2.5 g/L produces more driftable droplets than control and other SA solutions (Fig. 8). This is also evident from only marginal increase in size of DV_10_ category droplets (Table 9) when compared to control. Fig. 9 describing velocity of droplets from different treatments also exhibits the maximum velocity drop with SA at 2.5 g/L when compared to control. In summary, considering both the droplet size and velocity, it appears that the addition of SA to water make it more viscous but not heavier. Addition of SA to water significantly increases the droplet size but marginally reduces the droplet velocity. The increase in droplet size appears more dominant than the decrease in velocity and therefore addition of SA to water/water-based pesticide tank mix will probably reduce the off-target drift, which agrees well with the results obtained in the field experiments (Table 7, Table 8).

**Table 9:** Laboratory experiment results on droplet spectrum observed by the P15 Oxford Laser image analyzer for air induction flat fan nozzle AIXR 11002.

Treatment	Droplet size (μm)^a^			Proportion of droplets <150 μm
	DV_10_	DV_50_	DV_90_	
Water only Water and SA at 1.25 g/L Water and SA at 2.5 g/L Water and SA at 5.0 g/L	98 (86–110) 133 (121–145) 113 (101–125) 134 (122–146)	334 (301–367) 367 (334–400) 391 (359–424) 388 (355–421)	647 (467–828) 661 (480–841) 799 (618–979) 709 (529–890)	76 (73–78) 73 (70–75) 79 (76–81) 74 (72–76)

a 95 % confidence intervals shown in parentheses.

Some of our ongoing bioassay experiments with honeybees and typical insect pests of soybean and cotton indicate that SA is less toxic to honeybees than the widely used petroleum-based pesticide adjuvant PAM. The experimental results also suggest that SA does not interfere with targeted pest kill mechanisms (personal communication). In one of the recent studies, although SA was used to develop nanopesticide emulsions [53], to our knowledge, our study is the first to use SA as a drift-reducing pesticide adjuvant with the objective of protecting insect pollinators such as honeybees.

Our laboratory experiments and field application trials demonstrated the off-target drift reduction potential of SA. SA, which is compatible with some of the commonly used herbicides and insecticides that does not interfere with pesticide mechanisms to kill target crop pests and that does not have adverse effects on beneficial insects such as bees, has the potential to have an enormous positive impact on the pesticide adjuvant market. To protect the beekeeping industry and other pollinator species, research similar to these is currently needed. Also needed are some regulatory controls on pesticide adjuvants to protect beneficial insects such as honeybees.

### 3.8 Limitations of the study

There are several limitations of our study including that in the lab a simple backpack sprayer was used to study the effect of SA and PAM on spray droplets properties. Limited field experiments were carried out in cropped fields to estimate the drift reduction potential of SA. Although our ongoing study showed that compared to PAM, SA had less mortality rate of honey bees, more experiments are needed to test the adjuvant effect on other physiological parameters of honey bees.

### 3.9 Future directions

To test the safety for honey bees, studies placing hives near the fields will need to be performed to determine exposure of individual bees. Honey bees collected from within these hives will need to be analyzed for pesticide and adjuvant residue to determine exposure amounts. Testing multiple sprayer nozzles in a laboratory spray chamber setup will allow for comparison of adjuvants through different spray nozzles. Field trials for multiple crops with the algae-based adjuvant will need to be carried out using tractor-based booms to understand if the adjuvant efficiently delivers the pesticide to the target area. In addition, the ability of the adjuvant to make the pesticides (i) stick to the target surface and (ii) improve the rain fastness, droplet spread, and penetration will need to be analyzed.

## 4 Conclusions

Some adjuvants added to pesticide tank mix to improve spraying characteristics are equally/more toxic than the pesticide active ingredient. The health of bees raised in areas adjacent to cropped areas is affected by pesticide drift from cropped areas. Therefore, there is an urgent need to develop a pesticide adjuvant that is less toxic or nontoxic to insect pollinators and mitigates off-target pesticide drift. Our ongoing toxicological study of sodium alginate (SA) (an algae-based polymer) and polyacrylamide (PAM) (a petroleum-based adjuvant which is an industry standard) with honeybees and typical insect pests of soybean and cotton (using two widely used insecticides) provided encouraging results that pointed out reductions of in bee kills with SA as adjuvant and no interference in targeted pest kill mechanisms. Therefore, spray experiments were carried out in the laboratory and field to estimate the drift reduction capabilities of SA in comparison to PAM.

Based on our observations of droplet diameter from laboratory experiments, it can be interpreted that SA is able to reduce off-target drift when compared to no adjuvant control and is almost as good as PAM. When compared to the no adjuvant scenario, the addition of SA increases the droplet velocity only marginally. The drift reduction results exhibited by SA are less in terms of droplet velocity than in terms of droplet diameter. The results from all three field experiments collectively indicate reductions in off-target pesticide drift when SA is used as a pesticide adjuvant. In summary, SA can reduce pesticide drift similar to a commonly used commercial adjuvant and should be further developed as a next-generation pesticide adjuvant with equal efficacy and less toxicity to environment and pollinators.

The results described in this study provide the first baseline report of SA as an alternative adjuvant to ensure drift reduction and improve spraying characteristics, while being less toxic to honey bees. Improved spray technologies and chemicals such as SA can help improve the health of our pollinators and agro-ecosystems while ensure crop production to meet the growing global food demands.

## CRediT authorship contribution statement

**Narayanan Kannan:** Writing – original draft, Visualization, Project administration, Methodology, Formal analysis, Conceptualization. **Quentin Read:** Writing – original draft, Software, Formal analysis. **Weiqiang Zhang:** Writing – review & editing, Methodology, Data curation.

## Funding

This research is supported by internal funding from USDA-ARS under project number # 6066-30500-001-000D.

## Disclaimer

The findings and conclusions in this publication are those of the author(s) and should not be construed to represent any official USDA or U.S. government determination of policy.

## Declaration of competing interest

The authors declare that they have no known competing financial interests or personal relationships that could have appeared to influence the work reported in this paper.
