# Supplementary material for: An algae-based polymer material as a pesticide adjuvant for mitigating off-target drift
Source: Heliyon. 2024 Aug 2;10(16):e35510. doi: 10.1016/j.heliyon.2024.e35510 (PMC11357751; doi:10.1016/j.heliyon.2024.e35510)
Supplement: Multimedia component 1 [file mmc1.docx]

**
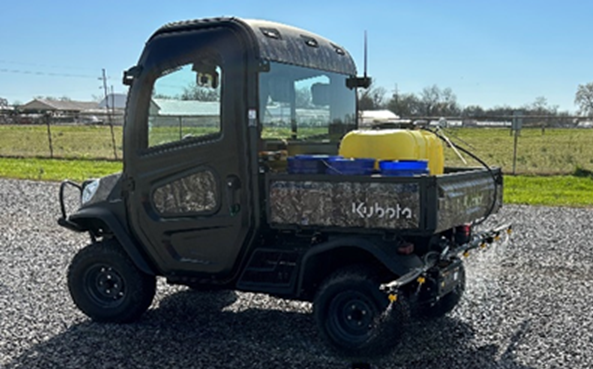
**

**Figure S1** Spraying equipment used in the field experiments.

**Table S1** Results of estimated means summarized by adjuvant dose.

|  | No adjuvant | Sodium alginate | Polyacryl-  amide | Sodium alginate | Polyacryl-  amide | Sodium alginate | Polyacryl-amide | Sodium alginate | Polyacryl-amide |
| --- | --- | --- | --- | --- | --- | --- | --- | --- | --- |
| Dose | None | All | All | Low | Low | Medium | Medium | High | High |
| Pesticide | Included | Included | Included | Included | Included | Included | Included | Included | Included |
| Nozzle | Both | Both | Both | Both | Both | Both | Both | Both | Both |
| Pressure (kPa) | All | All | All | All | All | All | All | All | All |
| Q_10_ | 100.5 (75.8- 127.2) | 105.9 (77.3- 136.5) | 118.4 (87.2- 151. 9) | 87.6 (63.7- 113.2) | 100.7 (76.1- 127.5) | 112.6 (82.7- 144.2) | 107.7 (82.7- 134.9) | 120.0 (87.4- 155.2) | 141.9 (92.6- 195.5) |
| Q_50_ | 154.3 (128.2- 188.5) | 176.8 (147.1- 215.9) | 193.5 (160.6- 236.1) | 148.8 (123.5- 181.8) | 154. 1 (128.2- 188.1) | 184.1 (152.8- 224.8) | 156.2 (130.0- 190.6) | 201.9 (167.3- 247.0) | 300.5 (246.9- 367.3) |
| Q_90_ | 236.5 (186.9- 318.7) | 295.1 (228.8- 410.4) | 315.5 (246.1- 436.4) | 252.4 (195.1- 352.9) | 235.4 (186.7- 316.4) | 300.5 (234.3- 415.6) | 226.3 (181.4- 300. 9) | 339.3 (261.9- 474.6) | 635.2 (463.5- 979.0) |
| Prop<150µm | 0.467 (0.241- 0.692) | 0.343 (0.163- 0.521) | 0.255 (0.092- 0.429) | 0.508 (0.320- 0.692) | 0.469 (0.242- 0.692) | 0.299 (0.125- 0.480) | 0.445 (0.196- 0.699) | 0.234 (0.081- 0.398) | 0.118 (0.028, 0.240) |

**Table S2** Results of estimated means summarized by spray pressure.

|  | No adjuvant | Sodium alginate | Polyacryl-  amide | Sodium alginate | Polyacryl-  amide | Sodium alginate | Polyacryl-amide | Sodium alginate | Polyacryl-amide |
| --- | --- | --- | --- | --- | --- | --- | --- | --- | --- |
| Dose | None | All | All | All | All | All | All | All | All |
| Pesticide | Included | Included | Included | Included | Included | Included | Included | Included | Included |
| Nozzle | Both | Both | Both | Both | Both | Both | Both | Both | Both |
| Pressure (kPa) | All | All | All | 131 | 131 | 172 | 172 | 200 | 200 |
| Q_10_ | 100.5 (75.8- 127.2) | 105.9 (77.3- 136.5) | 118.4 (87.2- 151. 9) | 123.6 (89.9- 159.4) | 134.9 (97.9- 174.6) | 97.3 (70.4- 125.6) | 112.6 (83.0- 144.8) | 98.6 (72.4- 126.9) | 109.0 (81.3- 139.3) |
| Q_50_ | 154.3 (128.2- 188.5) | 176.8 (147.1- 215.9) | 193.5 (160.6- 236.1) | 205.6 (170.5- 251.6) | 228.1 (189.3-278.7) | 165.4 (137.4- 202.0) | 183.4 (152.2- 224.2) | 162.5 (135.2- 198.5) | 173.0 (143.5- 210.8) |
| Q_90_ | 236.5 (186.9- 318.7) | 295.1 (228.8- 410.4) | 315.5 (246.1- 436.4) | 341.7 (265.5- 478.1) | 385.6 (298.2- 539.5) | 280.7 (216.6- 392.7) | 297.8 (232.0- 412.1) | 267.8 (208.2- 371.0) | 274.2 (215.8- 372.3) |
| Prop<150µm | 0.467 (0.241- 0.692) | 0.343 (0.163- 0.521) | 0.255 (0.092- 0.429) | 0.215 (0.070- 0.381) | 0.154 (0.037- 0.307) | 0.408 (0.226- 0.588) | 0.301 (0.122-0.483) | 0.420 (0.228- 0.612) | 0.349 (0.155- 0.552) |

**Table S3** Results of estimated means summarized by nozzle type.

|  | No adjuvant | Sodium alginate | Polyacryl-  amide | Sodium alginate | Polyacryl-  amide | No adjuvant | Polyacryl-amide | Sodium alginate | No adjuvant |
| --- | --- | --- | --- | --- | --- | --- | --- | --- | --- |
| Dose | None | All | All | All | All | All | All | All | All |
| Pesticide | Included | Included | Included | Included | Included | Included | Included | Included | Included |
| Nozzle | Both | Both | Both | Both | Both | Both | Both | Both | Both |
| Pressure (kPa) | All | All | All | Cone | Cone | Cone | Fan | Fan | Fan |
| Q_10_ | 100.5 (75.8- 127.2) | 105.9 (77.3- 136.5) | 118.4 (87.2- 151. 9) | 109.5 (79.7- 141.5) | 131.1 (98.0- 167.1) | 120.4 (92.7- 150.7) | 106.5 (77.0- 138.2) | 102.3 (75.0- 131.4) | 83.1 (61.0- 106.8) |
| Q_50_ | 154.3 (128.2- 188.5) | 176.8 (147.1- 215.9) | 193.5 (160.6- 236.1) | 184.1 (152.9- 224.7) | 205.3 (169.8- 250.6) | 173.4 (144.2- 212.1) | 182.3 (151.6- 222.1) | 169.7 (141.4- 207.4) | 137.2 (114.1- 167.6) |
| Q_90_ | 236.5 (186.9- 318.7) | 295.1 (228.8- 410.4) | 315.5 (246.1- 436.4) | 309.0 (239.5- 432.6) | 320.7 (252.6- 436.3) | 249.6 (199.9- 330.5) | 312.0 (240.4- 438.0) | 281.6 (219.0- 390.1) | 226.7 (176.5- 313.3) |
| Prop<150µm | 0.467 (0.241- 0.692) | 0.343 (0.163- 0.521) | 0.255 (0.092- 0.429) | 0.309 (0.137- 0.481) | 0.186 (0.045- 0.368) | 0.307 (0.096-0.559) | 0.323 (0.152- 0.489) | 0.379 (0.194- 0.564) | 0.589 (0.392- 0.778) |

**Table S4** Results of estimated means summarized by presence of pesticide.

|  | **No adjuvant** | **Sodium alginate** | **Polyacrylamide** |
| --- | --- | --- | --- |
| Dose | None | All | All |
| Pesticide | Included | Included | Included |
| Nozzle | Both | Both | Both |
| Pressure (kPa) | All | All | All |
| Q_10_ | 109.0 (83.1- 137.3) | 97.5 (70.3- 126.1) | 124.8 (90.4- 161.8) |
| Q_50_ | 161.0 (134.0- 196.4) | 167.0 (138.8- 204.0) | 211.6 (175.7- 258.6) |
| Q_90_ | 237.5 (189.5- 316.4) | 286.0 (220.9- 401.6) | 358.2 (277.3- 500.9) |
| Prop<150µm | 0.409 (0.177- 0.653) | 0.401 (0.220- 0.576) | 0.203 (0.064- 0.362) |


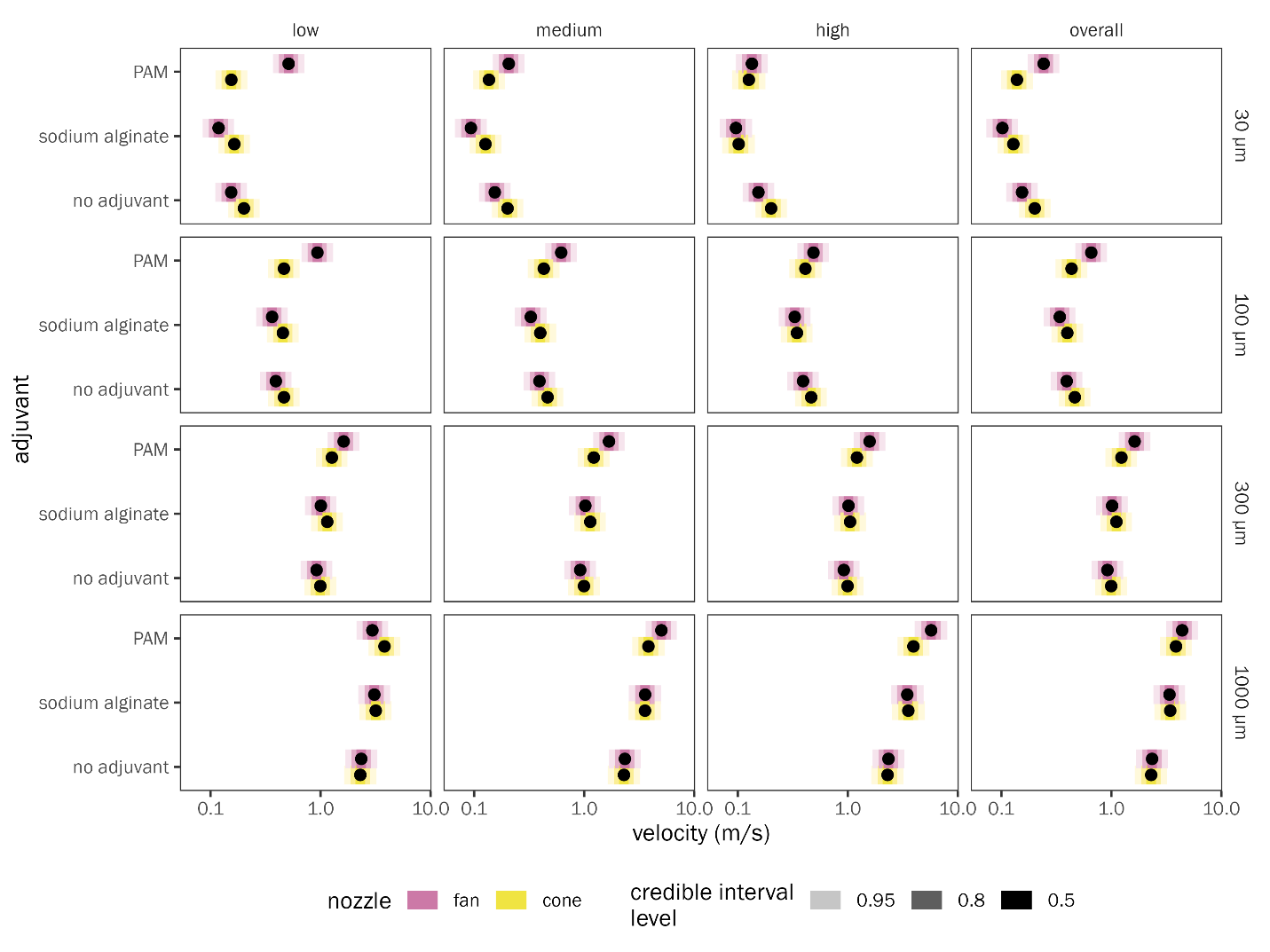


**Figure S2** Estimated marginal means of droplet velocity by adjuvant material and nozzle type, with each panel showing a different combination of adjuvant dose (low, medium, high, and averaged across all three dose levels) and particle size (30 µm, 100 µm, 300 µm, and 1000 µm). Points represent the median of the posterior distribution of the estimated marginal mean droplet velocity for each group, with progressively lighter-shaded error bars representing 50%, 80%, and 95% quantile credible intervals around the point estimates.


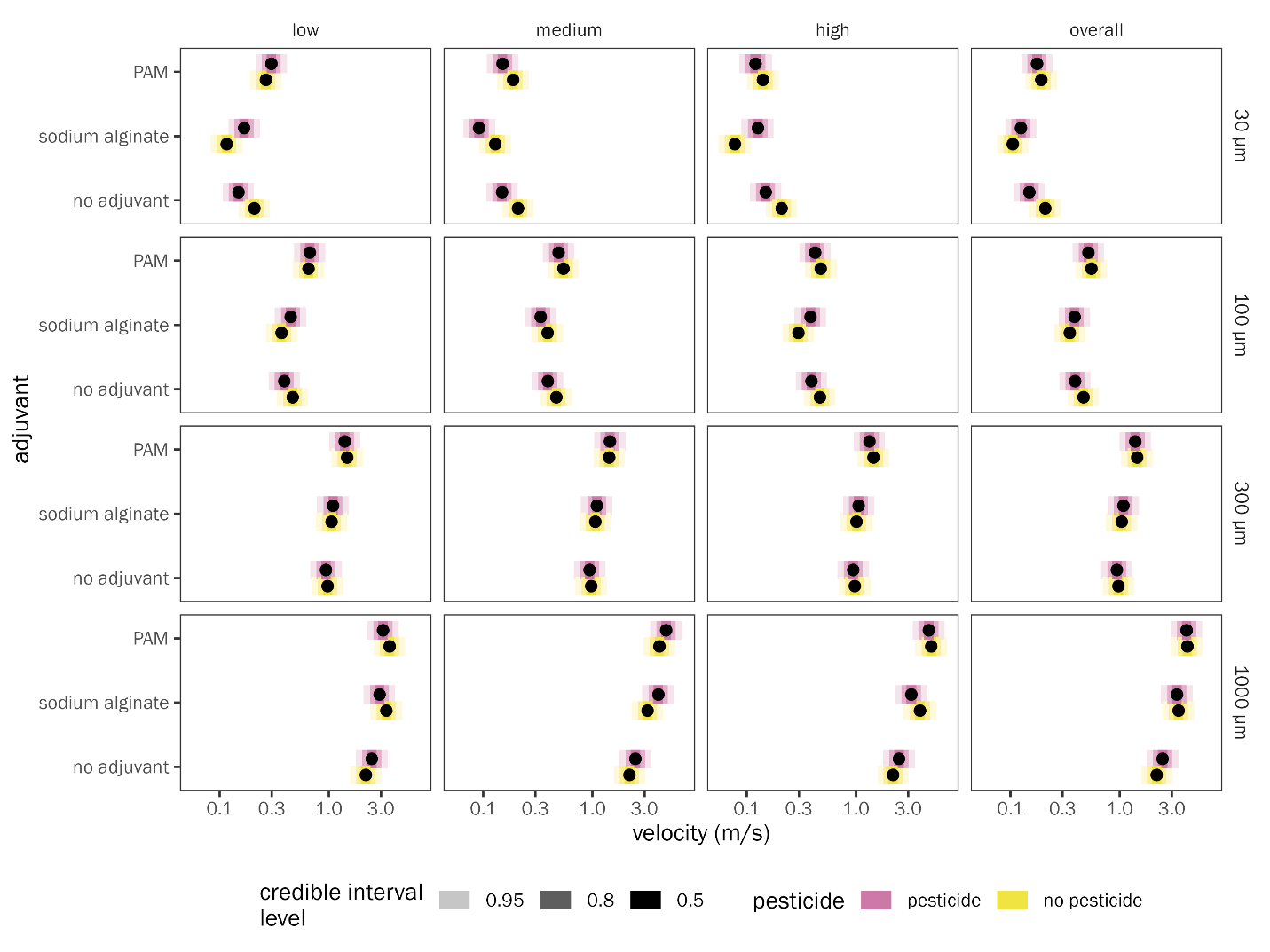


**Figure S3** Estimated marginal means of droplet velocity by adjuvant material and presence of pesticide, with each panel showing a different combination of adjuvant dose (low, medium, high, and averaged across all three dose levels) and particle size (30 µm, 100 µm, 300 µm, and 1000 µm). Points represent the median of the posterior distribution of the estimated marginal mean droplet velocity for each group, with progressively lighter-shaded error bars representing 50%, 80%, and 95% quantile credible intervals around the point estimates.


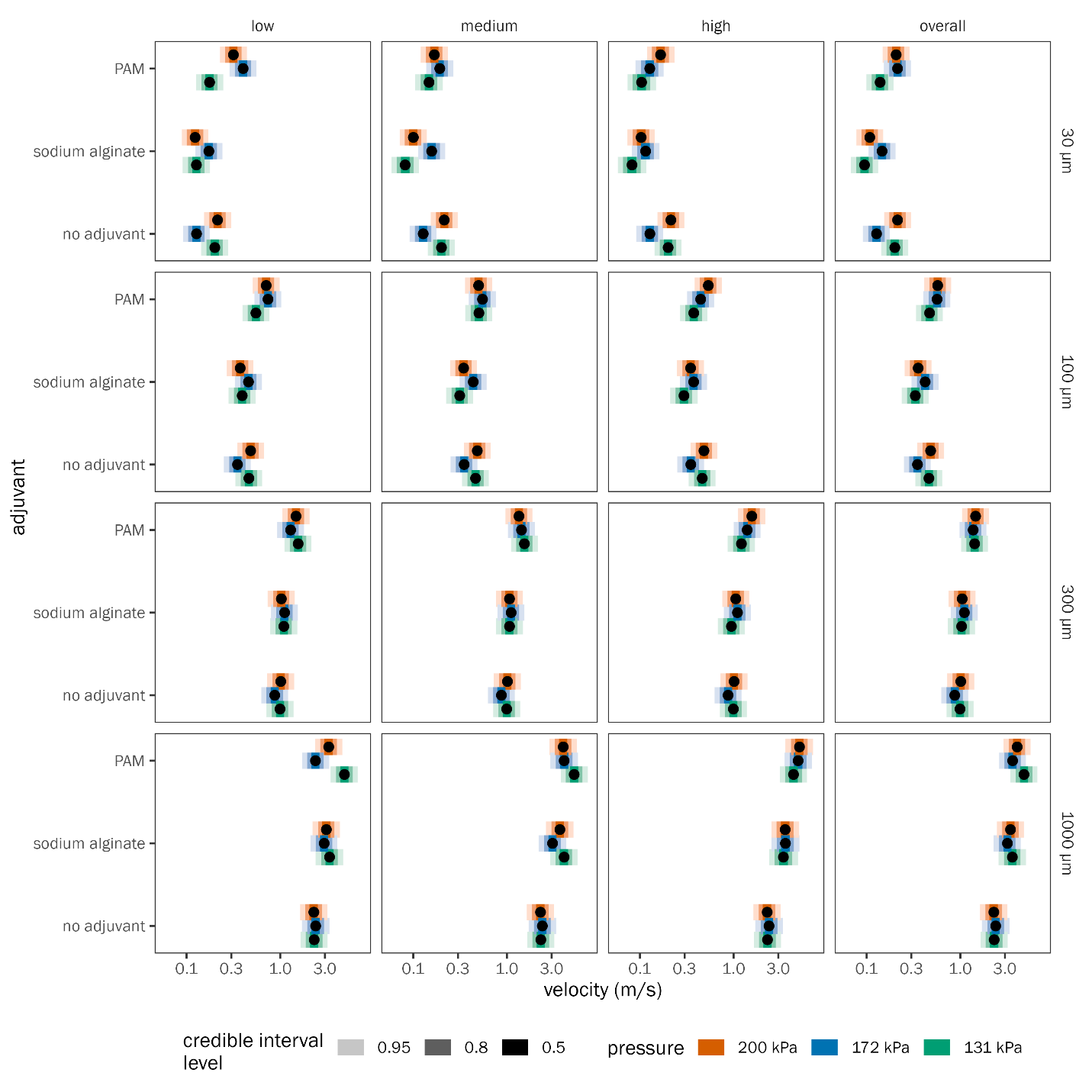


**Figure S4** Estimated marginal means of droplet velocity by adjuvant material and spraying pressure, with each panel showing a different combination of adjuvant dose (low, medium, high, and averaged across all three dose levels) and particle size (30 µm, 100 µm, 300 µm, and 1000 µm). Points represent the median of the posterior distribution of the estimated marginal mean droplet velocity for each group, with progressively lighter-shaded error bars representing 50%, 80%, and 95% quantile credible intervals around the point estimates.
